# Identification of *HXK* Gene Family and Expression Analysis of Salt Tolerance in *Buchloe dactyloides*

**DOI:** 10.3390/ijms26020838

**Published:** 2025-01-20

**Authors:** Haole Qi, Sining Wang, Yuehan Liu, Xueping Wang, Xiaoxia Li, Fengling Shi

**Affiliations:** 1 Key Laboratory of Grassland Resources of the Ministry of Education, College of Grassland Science, Inner Mongolia Agricultural University, Hohhot 010010, China; m19969045212@163.com; 2 Institute of Ecological Conservation and Restoration, Chinese Academy of Forestry, Grassland Research Center, National Forestry and Grassland Administration, Beijing 100091, China; wangsining66@163.com (S.W.); xjhbhlyh@163.com (Y.L.); lixiaoxia@caf.ac.cn (X.L.); 3 College of Pratacultural Science, Gansu Agricultural University, Lanzhou 730070, China; 18919325302@163.com

**Keywords:** *Buchloe dactyloides*, *HXK* gene family, salt stress, differential expression analysis, correlation network

## Abstract

*Buchloe dactyloides* is one of the typical ecological grass species, characterized by its strong salt tolerance. Hexokinase (HXK) plays a crucial role in plant growth, development, and resistance to abiotic stresses. To understand the function of *HXKs* in the salt tolerance of *B. dactyloides*, this study identified and analyzed the *HXK* gene family members using the whole-genome data of *B. dactyloides*. Additionally, transcriptomic methods were employed to investigate the expression levels and stress response patterns of the *HXK* family genes under salt stress. The results showed that 25 *HXK* genes were identified in the *B. dactyloides HXK* gene family, which were classified into three subfamilies based on the phylogenetic tree. Members within the same subfamily exhibited similar gene structures and conserved motifs. The promoter regions of *BdHXKs* contained numerous cis-regulatory elements associated with plant hormone responses, plant growth and development, and resistance to abiotic stresses. Quantitative real-time PCR analysis provided preliminary evidence that the *BdHXK5*, *BdHXK7*, and *BdHXK23* genes might play important roles in the salt tolerance regulation of *B. dactyloides*. These findings offer a theoretical foundation for further elucidating the functions and molecular regulatory mechanisms of *BdHXKs* under salt stress. This study has provided a theoretical basis for the breeding of new varieties of ecological restoration grasses with stronger salt tolerance and better growth and development. This is of great significance for the improvement and ecological restoration of saline–alkali land.

## 1. Introduction

*Buchloe dactyloides* (Nutt.) Engelm., also known as buffalo grass or buffalograss, is a perennial herbaceous plant belonging to the genus *Buchloe* in the subfamily *Eragrostoideae* of the family *Gramineae*. It is native to the semi-arid temperate and subtropical regions of central North America [1]. Compared to most turfgrasses, *B. dactyloides* exhibits remarkable traits such as tolerance to poor soil, cold resistance, drought resistance, and salt–alkali tolerance, along with extremely low maintenance costs, making it an important ecological grass species for improving saline–alkali soils and a highly promising candidate for development [2].

In recent years, global climate change and the frequent occurrence of natural disasters have increasingly drawn attention to the problem of soil salinization. According to statistics from UNESCO and the Food and Agriculture Organization of the United Nations, the global area of saline–alkali land is approximately 954.38 million hectares [3]. In China, the scale of saline–alkali land is also substantial, covering about 99.13 million hectares, and these figures continue to grow [4]. Soil salt stress can disrupt the normal physiological metabolism of plants through osmotic stress, ion stress, and oxidative stress, thereby affecting plant growth and development and even causing plant death [5]. To resist salt stress, plants employ a series of complex physiological activities and metabolic processes as defense mechanisms.

Hexokinase (HXK) is ubiquitously present in various plants and primarily participates in the phosphorylation of hexoses. By regulating glycolysis, it provides substrates for other physiological processes, thereby influencing plant growth and development [6]. *HXK* genes have been cloned in several plant species, including *Arabidopsis thaliana* [7], rice [8], corn [9], tomato [10], and *Sorghum bicolor* [11], and all findings indicate their significant role in sugar metabolism [12]. Furthermore, HXK can function as a regulatory signal. For instance, *PsHXK1* and *PsHXK2* can enhance glucose sensitivity, leading to the accumulation of anthocyanins [13]; *GcHXK* in citrus can induce stomatal closure, reducing the photosynthetic rate and affecting seed germination [14]. Many studies have confirmed that *HXK* is also involved in biotic and abiotic stress responses. Under salt stress, hexokinase catalyzes the phosphorylation of hexoses, providing energy and products that participate in osmotic regulation and antioxidant defense, helping plants withstand the damage caused by salt stress. *PrunusHXK3* can enhance the salt and drought tolerance of *Arabidopsis* transgenic plants by altering primary C-metabolism [15]; the overexpression of *GmHXK15* significantly enhances the tolerance of transgenic soybeans to alkaline stress [16]; *CsHXKs* genes in tea plants play a critical role in responding to four types of abiotic stresses: drought, salinity, cold, and exogenous abscisic acid (ABA) [17]. Therefore, HXK is considered a bifunctional enzyme, not only catalyzing metabolic reactions such as glucose phosphorylation but also serving as a key connecting element between sugar signaling and plant hormone signaling, playing a vital role in plant growth and development and environmental adaptation [18].

Despite the important functions of *HXK* genes, there are no reported studies on the *HXK* gene family in *B. dactyloides*. This study, based on the whole-genome data of *B. dactyloides* (the genome of the cultivar homologous tetraploid was made available in high quality in 2024), identified and analyzed 25 members of the *HXK* gene family. It included predictions of protein physicochemical properties, chromosomal localization, cis-acting element analysis, motif prediction, and analysis of conservation motifs. Additionally, the expression patterns of these genes in different tissues under salt stress were investigated. The aim is to provide a theoretical foundation for subsequent research on the functions of *HXK* genes and for the genetic improvement of salt tolerance using these genes.

## 2. Results

### 2.1. Identification and Physicochemical Property Analysis of B. dactyloides HXK Family Members

Based on the HXK sequences from *Arabidopsis thaliana*, rice (*Oryza sativa*), and *Brachypodium distachyon*, a Blast search was conducted on the full-length genome data of *B. dactyloides*. A total of 25 *HXK* genes were identified and named *BdHXK1* to *BdHXK25* (Supplementary Table S1). The physicochemical properties of these 25 *BdHXKs* genes were analyzed using the MEME online tool. The amino acid sequence lengths ranged from 441 residues (*BdHXK1*) to 531 residues (*BdHXK23*). The molecular weights varied from 47,372.95 Da (*BdHXK1*) to 58,056.96 Da (*BdHXK23*). As plants adapt to their environment, they develop unique metabolic requirements. This leads to the mutation and duplication of encoding genes, which in turn alters the protein structure and thus affects the molecular weight. The theoretical isoelectric points (pI) ranged from 5.03 to 6.02, indicating that all *BdHXKs* family proteins are acidic and rich in acidic amino acids. This is somewhat similar to what is observed in some grasses, such as *Setaria italica*, where many hexokinases also have acidic pI values [19]. The instability index of *BdHXKs* genes ranged from 33.41 to 46.38, suggesting that these genes are relatively stable. The aliphatic index was between 89.65 and 103.84, and the hydropathy index of *BdHXKs* genes ranged from -0.172 to 0.167. *BdHXK2*, *BdHXK3*, *BdHXK8* to *BdHXK13*, and *BdHXK22* to *BdHXK25* had positive hydropathy indices, indicating that they are likely hydrophobic proteins. In contrast, *BdHXK1*, *BdHXK4* to *BdHXK7*, and *BdHXK14* to *BdHXK21* had negative hydropathy indices, suggesting that they are hydrophilic proteins. Subcellular localization predictions indicated that BdHXK6, BdHXK7, BdHXK18, and BdHXK21 are located in the chloroplast, while BdHXK2, BdHXK3, BdHXK8 to BdHXK10, BdHXK19 to BdHXK20, and BdHXK22 to BdHXK25 are located in the mitochondria. The remaining proteins were found to be predicted in both chloroplasts and mitochondria. Environmental factors drive the evolution of HXK in plants toward different physicochemical properties to respond to diverse ecological niches. For example, under salt stress, *B. dactyloides* generates HXK with enhanced stability. This ensures that effective glycolysis can provide energy to counteract ion toxicity. Meanwhile, as salt stress causes water efflux from cells, the hydrophilicity of HXK increases. This promotes sufficient substrate–enzyme interactions, thus helping the plant maintain growth under osmotic stress.

### 2.2. Chromosomal Localization and Gene Structure Analysis of the B. dactyloides HXK Gene Family

**Figure 1:**
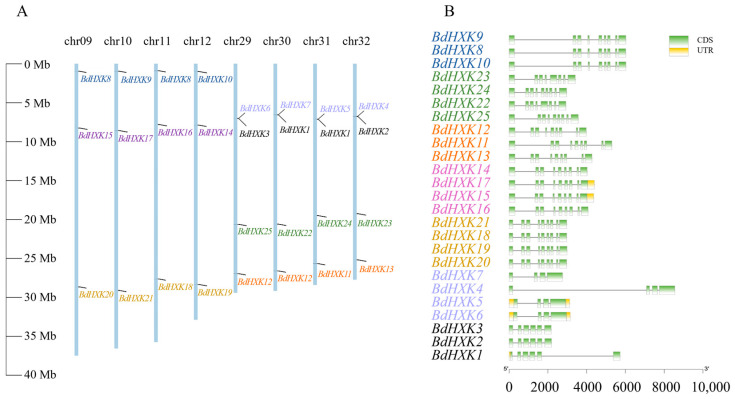
Chromosome distribution and gene structure of *HXK* family genes in *Buchloe dactyloides.* (**A**) A total of 8 chromosomes with varying lengths are shown in relation to the Mb (million base pair) scale on the left, and individual chromosomes (bars) are labeled with respective *BdHXK* genes. (**B**) Genetic structure analysis of *BdHXK*s gene family members, with exons represented by yellow rectangles and introns represented by black lines.

The chromosomal positions of *BdHXKs* genes were determined using TBtools software. *Buchloe dactyloides* has a total of 40 chromosomes. Chromosomes 9, 10, 11, and 12 each contained three *BdHXK* genes, and their distribution positions were similar. Chromosomes 29, 30, 31, and 32 each contained four *BdHXKs* genes, with similarly distributed positions. *BdHXK3* and *BdHXK6*, *BdHXK1* and *BdHXK7*, *BdHXK1* and *BdHXK5*, and *BdHXK2* and *BdHXK4* were clustered in small regions (Figure 1A). These results indicate that *BdHXK* gene family members are unevenly distributed across the chromosomes, suggesting that gene tandem duplication events may have occurred during evolution, which could facilitate the rapid response of *B. dactyloides* to salt stress and enhance its salt tolerance. Our gene structure analysis of *BdHXK* genes using TBtools software (Figure 1B) showed that the structures of genes at the same positions were highly similar. Each gene contained three to eight introns. The number of exons in different genes ranged from 4 to 9, with 4 genes (16% of the total) containing 4 exons, 3 genes (12% of the total) containing 6 exons, 2 genes (8% of the total) containing 8 exons, and 16 genes (64% of the total) containing 9 exons.

### 2.3. Phylogenetic Tree and Conserved Motif Analysis of the B. dactyloides HXK Gene Family

**Figure 2:**
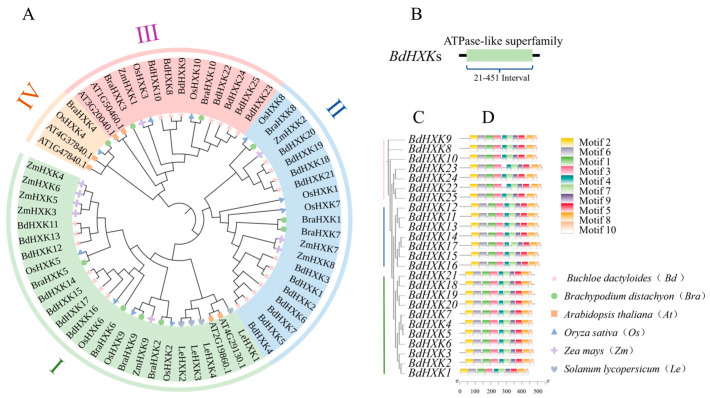
Phylogenetic trees, conserved domain, and conserved motif of *HXK* family members for *Buchloe dactyloides*. (**A**) Phylogenetic tree was constructed based on *HXK* sequences of *Buchloe dactyloides*, *Arabidopsis thaliana*, *Oryza sativa*, *Zea mays*, *Solanum lycopersicum*, and *Brachypodium distachyon*. The tree was then categorized into four groups, each represented by a distinct color. (**B**) Conserved domain analysis of *Buchloe dactyloides HXK* gene family members. (**C**) Evolutionary relationship of *HXK* gene family in *Buchloe dactyloides*. (**D**) A total of 10 motifs were identified, represented by rectangles of different colors.

To further understand the evolutionary relationships of *BdHXKs* genes with *HXK* genes in other species, a phylogenetic tree was constructed by aligning 6 *HXK* genes from *Arabidopsis thaliana*, 10 *HXK* genes from rice (*Oryza sativa*), 9 *HXK* genes from *Zea mays*, 4 *HXK* genes from *Solanum lycopersicum*, and 10 *HXK* genes from *Brachypodium distachyon* with the 25 *HXK* genes identified in *B. dactyloides* (Figure 2A). The results indicate that the 25 *BdHXKs* genes have high homology with those from rice and *Brachypodium distachyon*, but a relatively distant relationship with those from *Arabidopsis thaliana*. Based on their homology with rice *HXK* genes, the *BdHXKs* genes were divided into three phylogenetic clusters [20]: Cluster I: *BdHXK11* to *BdHXK17* and *OsHXK2*, *OsHXK5*, *OsHXK6*, and *OsHXK9*; Cluster II: *BdHXK1* to *BdHXK7* and *BdHXK18* to *BdHXK21* with *OsHXK1*, *OsHXK7*, and *OsHXK8;* Cluster III: The remaining *BdHXK* genes and *OsHXK3* and *OsHXK10*; Cluster IV: None of the *BdHXK* genes were clustered with *OsHXK4*.

Furthermore, the conserved domains of the *BdHXKs* gene family members were analyzed using the NCBI conserved domain search program (Figure 2B). The results show that *BdHXK* proteins contain the ASKHA ATPase-like superfamily domain, which is generally located between residues 21 and 451. Proteins within the same subfamily share similar conserved domains, indicating that genes in the same subfamily have a relatively conserved evolution and may have similar regulatory mechanisms and functions. Studies have shown that these proteins can accelerate sugar metabolism by catalyzing the phosphorylation of glucose, fructose, and sorbitol, producing more ATP to meet cellular energy requirements and enhance salt tolerance [21]. In some plants, hexokinases can also regulate the activity of sodium-potassium ion exchange factors, promoting the efflux of sodium ions from cells and maintaining ion balance, thereby enhancing plant salt tolerance [21].

To further identify the conserved motifs of the *BdHXKs* proteins, the MEME website was used to predict the conserved motifs of the encoded proteins, and 10 conserved motifs were identified (Figure 2D). Combining the phylogenetic results of the *BdHXKs* genes (Figure 2C), it is evident that most family members contain all 10 motifs, and their arrangement order is also largely the same. This suggests that the *BdHXK* gene family members have similar and conserved structures and may play similar roles in salt tolerance regulation in *B. dactyloides*.

### 2.4. qRT-PCR Validation Analysis of the B. dactyloides HXK Family Under Salt Stress

**Figure 3:**
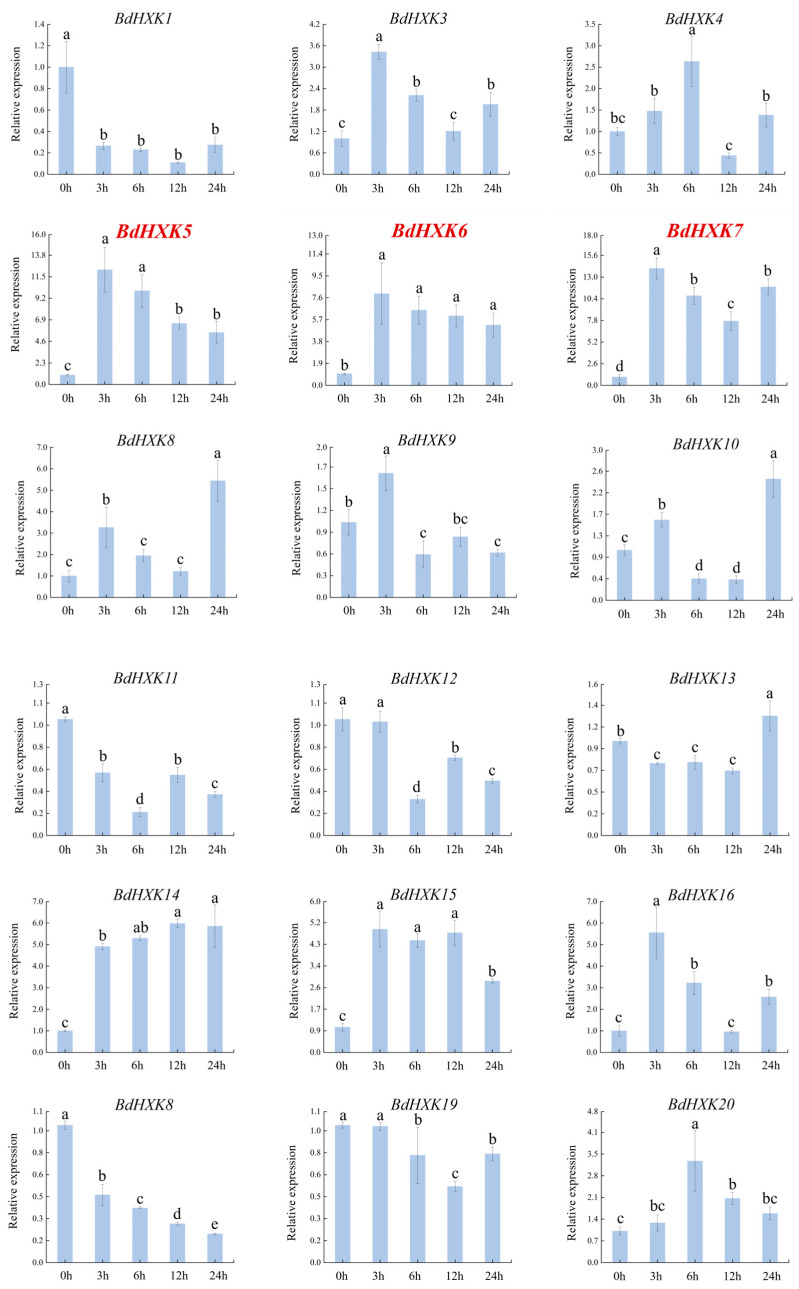

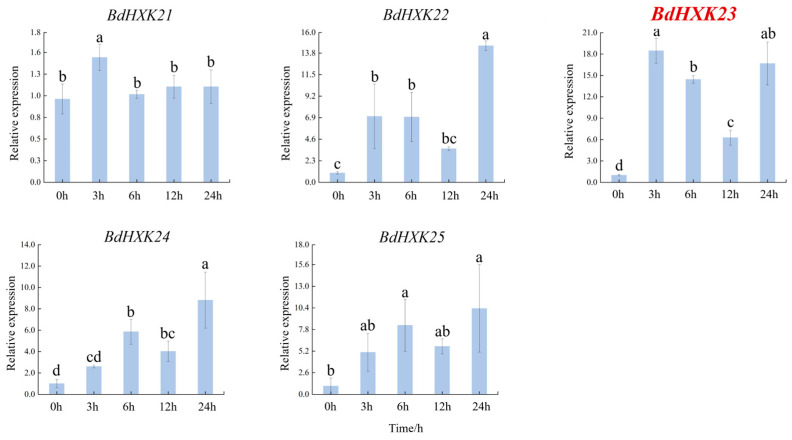
Expression pattern of *HXK* gene in *Buchloe dactyloides* under salt stress. The horizontal coordinate is the processing time, and the vertical coordinate is the relative expression level. Duncan’s test of SPSS was used to determine the significance of the differences. Different letters indicate significant differences between groups (*p* < 0.05).

To investigate the response of *HXK* genes to salt stress, the differential expression of *BdHXKs* genes of *B. dactyloides* under 600 mM NaCl stress was analyzed using real-time quantitative PCR (RT-qPCR). The RT-qPCR results (Figure 3) show the following expression patterns: *BdHXK5*, *BdHXK6*, *BdHXK14*, and *BdHXK20*: their relative expression levels first increased and then decreased; *BdHXK9*, *BdHXK15*, and *BdHXK21*: their relative expression levels first increased, then decreased, and showed a slight recovery at 12 h; *BdHXK3*, *BdHXK4*, *BdHXK7*, *BdHXK8*, *BdHXK10*, *BdHXK16*, *BdHXK22*, *BdHXK23*, *BdHXK24*, and *BdHXK25*: their relative expression levels first increased and then decreased, with a recovery at 24 h; *BdHXK1*, *BdHXK13*, and *BdHXK19*: their relative expression levels first decreased and then increased; *BdHXK11* and *BdHXK12*: their relative expression levels first decreased, then increased, and showed a decline again at 24 h; *BdHXK18*: it exhibited a unique pattern, showing continuous decline without any recovery; *BdHXK2* and *BdHXK17*: they did not show significant expression changes under salt stress.

Among these, *BdHXK5*, *BdHXK6*, *BdHXK7*, and *BdHXK23* were more sensitive to salt stress, and their relative expression levels were significantly upregulated, making them potential candidates for further research on salt tolerance in *B. dactyloides*.

### 2.5. Tissue-Specific Expression Analysis of Key B. dactyloides HXK Genes

**Figure 4:**
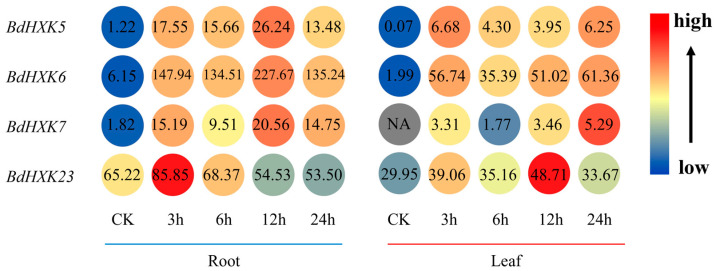
Expression differential of *Buchloe dactyloides HXK* key gene in different tissues. The expression profiles of the *BdHXK5*, *BdHXK6*, *BdHXK7*, and *BdHXK23* genes under different tissues and different durations of salt stress treatment are presented in a heatmap. In the heatmap, the color ranging from blue to red indicates the expression levels from low to high, with the specific expression amounts shown as numerical values. On the left side is the expression level of the key genes in roots at different periods, and on the right side is the expression level in leaves at different periods.

Transcriptome data were used to analyze the expression of *HXK* genes in the roots and leaves of *B. dactyloides* (Figure 4). The results show that the expression levels of these genes vary across different tissues. *HXK* genes exhibit higher expression in the roots compared to the leaves, where the expression levels are significantly reduced. Among the four key genes, their expression levels were significantly upregulated at 3 h under salt stress. In the roots, the expression level of *BdHXK6* was notably higher than that of the other genes, suggesting that it may play a crucial role in salt stress tolerance.

### 2.6. Promoter Analysis of Key Members of the B. dactyloides HXK Gene Family

Analysis of the cis-acting elements in the promoters of *B. dactyloides* can provide insights into the tissue-specific or stress-responsive expression patterns of these genes. As shown in Supplementary Figure S1, the promoters of each *BdHXK* gene generally contain different types of plant hormone response elements, abiotic stress response elements, and plant growth and development response elements. The plant hormone response elements include 10 types: abscisic acid response elements (ABRE), methyl jasmonate response elements (CGTCA-motif and TGACG-motif), auxin response elements (TGA-element and AuxRR-core), gibberellin response elements (P-box and TATC-box), zein metabolism regulation elements (O2-site), and salicylic acid response elements (TCA-element). The abiotic stress response elements include six types: anoxic-inducible cis-elements (ARE), hypoxia-specific induction enhancers (GC-motif), MYB drought-induced binding sites (CCAAT-box and MBS), low-temperature response cis-elements (LTR), and cis-acting factors involved in salt stress defense and response (TC-rich repeats). Finally, the plant growth and development response elements include 15 types: light response elements (G-box, AAAC-motif, ATCT-motif, Box4, Box II, GA-motif, GT1-motif, I-box, Pc-CMA2c, Sp1, TCCC-motif, and TCT-motif), meristematic tissue expression regulation elements (CAT-box), and seed-specific regulation cis-elements (RY-element).

Each of the 25 *BdHXKs* genes contains 11 to 20 different cis-acting elements, with significant variations in the types and quantities of these elements. All members of the *BdHXKs* gene family contain light response, abscisic acid, and methyl jasmonate-related cis-acting elements, which are significantly associated with plant abiotic stress tolerance. This suggests that the *BdHXKs* gene family in *B. dactyloides* has diverse functions and may play important roles in plant growth and development as well as in resistance to salt, drought, low temperature, and anoxic stresses. Notably, some members, particularly *BdHXK6*, contain unique cis-acting elements such as TC-rich repeats, TCA-element, and ARE, which can interact with specific genes to regulate the expression of downstream genes, thereby influencing the defense and stress response of *B. dactyloides* to salt stress.

**Figure 5:**
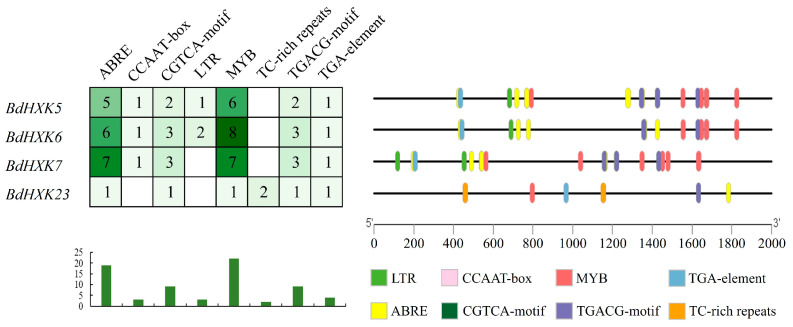
*Buchloe dactyloides HXK* key gene cis-acting element. Different cis-acting elements are represented in different colors. These cis-acting elements may be related to plant stress resistance and hormone regulation.

Promoter analysis of the key genes *BdHXK5*, *BdHXK6*, *BdHXK7*, and *BdHXK23* was conducted, and the results are shown in Figure 5. *BdHXK6* contains the highest number of various cis-acting elements, while *BdHXK23* has the fewest, lacking the CCAAT-box and LTR elements but containing the unique TC-rich repeats element. Statistical analysis revealed that abscisic acid, MYB drought-induced binding sites, and methyl jasmonate-related cis-acting elements are the most prevalent. Research has shown that abscisic acid is significantly associated with plant abiotic stress tolerance, and methyl jasmonate is related to plant growth and development, as well as abiotic stress tolerance. This indicates that *BdHXK5*, *BdHXK6*, *BdHXK7*, and *BdHXK23* may play strong roles in plant growth and development and in responses to abiotic stress.

### 2.7. Correlation Network Analysis

**Figure 6:**
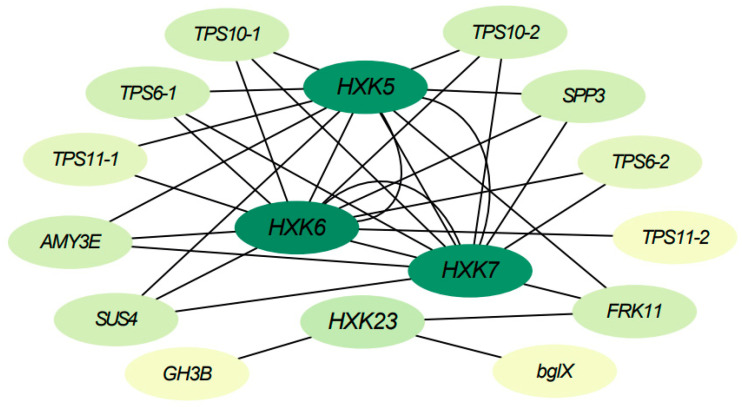
*Buchloe dactyloides BdHXK5*, *BdHXK6*, *BdHXK7*, and *BdHXK23* genes’ correlation network analysis in leaves and roots. The ovals represent each gene. The darker the color, the higher the number of interactions with other genes. Edges represent interactions between genes.

To further understand the functional mechanisms of *BdHXKs* genes, a correlation network analysis was performed based on transcriptome data for *BdHXK5*, *BdHXK6*, *BdHXK7*, and *BdHXK23*. The results are presented in Figure 6, and detailed information on the interacting genes is provided in Supplementary Table S3. The interaction gene information shows that *BdHXK5*, *BdHXK6*, *BdHXK7*, and *BdHXK23* are significantly positively correlated with genes from families such as trehalose-6-phosphate synthase (TPS), sucrose phosphate synthase (SPP), α-amylase (AMY), and sucrose synthase (SUS). This suggests the possibility of upstream and downstream interactions between these genes. Moreover, the same *BdHXK* gene can interact with multiple other *BdHXK* genes, indicating a tight interaction network within the *BdHXK* gene family. These findings suggest that *HXK* family genes in *B. dactyloides* may regulate various metabolic processes through interactions with genes involved in carbohydrate supply and conversion, providing more energy and carbon sources to the plant, helping to maintain osmotic balance in plant cells, and reducing cell dehydration caused by salt stress, thereby enabling the plant to better cope with salt stress conditions.

## 3. Discussion

Hexokinase (HXK) not only plays a crucial role in plant metabolism but is also actively involved in the response to various abiotic stresses such as salinity, alkalinity, and drought. To date, 6 *HXK* members have been reported in *Arabidopsis thaliana* [7], 10 members in rice (*Oryza sativa*) [8], 9 members in *Zea mays* [9], 4 members in *Solanum lycopersicum* [10], and 10 members in *Brachypodium distachyon*. However, no studies have reported *HXK*s in *Buchloe dactyloides*. In this study, based on the *B. dactyloides* genome database, 25 members of the *HXK* gene family were identified, which is significantly more than in other plants. The *HXK* gene family plays an important role in the growth, development, and environmental adaptation of *B. dactyloides*. In the long-term evolutionary process, *B. dactyloides* may face specific natural selection pressure, making the expansion of the *HXK* gene family a favorable evolutionary strategy. Therefore, we speculate that the number of *HXK* gene family members may increase in order to adapt to the environment such as salt stress, so as to better regulate various physiological processes.

In previous research, we comprehensively applied flow cytometry and the chromosome squash method and found that *B. dactyloides* had multiple ploidies [22]. We selected the most representative tetraploid materials for in-depth study. As a homologous tetraploid plant, *B. dactyloides* presents a unique gene distribution pattern in its genetic architecture. Specifically, on the homologous chromosomes numbered 9, 10, 11, and 12, each chromosome carries three members of the *HXK* gene family, and on the homologous chromosomes numbered 29, 30, 31, and 32, each chromosome has four members of the *HXK* gene family. Through in-depth research, it was found that there were differences in the sequences of alleles on homologous chromosomes. Therefore, the proteins encoded by them were also different, and it was further speculated that there would also be differences in their functions. Hence, we determined that these genes were different gene individuals. This phenomenon was most likely the result of natural selection during the long evolutionary process. Previous studies have shown that most *HXK* genes in model plants such as rice, *Arabidopsis*, foxtail millet, and sorghum contain nine exons [23]. In our study, 16 of the 25 *BdHXKs* genes contained 9 exons, while the remaining members had fewer exons due to intron loss, containing 4, 6, or 8 exons. This indicates that the gene structures of different *BdHXK* members are highly variable. By studying the structural changes, we can infer the evolutionary history of the gene family. Current research on *HXK* genes in rice and *Arabidopsis* suggests that they originated from a common ancestor [20]. Given the close evolutionary relationships between *B. dactyloides* and rice, *Arabidopsis*, and *B. distachyon*, it is reasonable to hypothesize that *B. dactyloides* may have evolved from the same ancestor.

Through the construction and analysis of a phylogenetic tree for *BdHXKs* gene in *B. dactyloides*, *Arabidopsis thaliana*, *Oryza sativa*, *Zea mays*, *Solanum lycopersicum*, and *Brachypodium distachyon*, *BdHXKs* can be clearly divided into three subfamilies, consistent with previous findings in rice [20]. Each *BdHXK* gene in *B. dactyloides* has at least one homologous gene in rice and *B. distachyon*. For example, *BdHXK1* to *BdHXK7* share the closest evolutionary relationship with *OsHXK1*, suggesting that they may regulate sugar signal transduction and interact with other signaling molecules to maintain ion balance inside and outside cells [24]. *BdHXK14* is most closely related to *OsHXK6* in rice and *BdHXK6* in *B. distachyon*, indicating that it may be light-induced and have similar functions, potentially contributing to the plant’s response to abiotic stress, such as by regulating sugar metabolism and energy supply to help *B. dactyloides* adapt to high-salinity environments and maintain normal cellular physiological functions [25].

Subcellular localization analysis of *BdHXKs* family members revealed that BdHXK6, BdHXK7, BdHXK18, and BdHXK21 are specifically located in chloroplasts, where they can phosphorylate hexoses or bind to the chloroplast membrane to phosphorylate glucose [26]. Notably, *BdHXK7* showed a significant upregulation under salt stress, with its expression level increasing by up to 14-fold. This suggests that *BdHXK7* may accelerate the catalysis of sugar metabolism under salt stress, providing energy for the synthesis of osmotic regulatory substances such as proline and antioxidant enzymes [27]. BdHXK2, BdHXK3, BdHXK8, BdHXK9, BdHXK10, BdHXK19, BdHXK20, and BdHXK22 to BdHXK25 are specifically located in mitochondria, possibly through membrane-anchored structures, and may contribute to the regulation of plant sugar signaling [26]. Other BdHXK proteins are expressed in both chloroplasts and mitochondria, collectively participating in the regulation of sugar metabolism and plant growth and development.

HXK plays an irreplaceable role in the normal growth, development, and metabolic activities of organisms [28]. Multiple studies have shown that HXK, in response to salt stress, primarily catalyzes the phosphorylation of hexoses, converting them into products like glucose-6-phosphate, thereby initiating metabolic pathways such as glycolysis to provide cells with sufficient energy to cope with the stress caused by salinity [29]. Additionally, HXK can regulate the activity of sodium and hydrogen ion exchangers through phosphorylation, promoting the efflux of sodium ions from cells to maintain the intracellular ion balance [30]. For example, *MdHXK1* in apples interacts with the Na^+^/H^+^ exchanger and phosphorylates the Ser275 residue, enhancing the stability and transport activity of *MdNHX1*, thus improving the salt tolerance of apple plants [31].

The differential expression analysis revealed that the expression levels of the remaining *BdHXK* members varied, indicating that different members of the *HXK* gene family play distinct roles in the plant’s response to salt stress. Notably, *BdHXK5*, *BdHXK6*, *BdHXK7*, and *BdHXK23* showed a significant upregulation of up to 14–18-fold under salt stress, making them potential candidate genes for further functional studies on salt stress response in *B. dactyloides*. Subsequently, we analyzed the expression patterns of *BdHXK5*, *BdHXK6*, *BdHXK7*, and *BdHXK23* genes in the roots and leaves of *B. dactyloides* through transcriptome data. The research results revealed that the expression levels of these genes in the roots were significantly higher than those in the leaves. The *HXK* gene family demonstrated the characteristic of high expression in root tissues. As the root is the main salt-sensing organ of plants, this finding implies that the *HXK* genes may play a significant role in the process of *B. dactyloides* responding to salt stress.

Further analysis of the cis-acting elements in the promoter regions of key *BdHXKs* genes in *B. dactyloides* revealed that most of them contain stress-responsive elements such as ABRE, ARE, and TC-rich repeats [32]. This suggests that the *BdHXKs* gene family may play important roles in plant growth and development, as well as in resistance to abiotic stresses like salinity, drought, low temperature, and anoxia.

Transcriptome correlation network analysis revealed a significant positive correlation between key *BdHXK* members and genes from families such as *TPS*, *SPP*, *AMY*, and *SUS*, suggesting potential upstream and downstream interaction relationships. For instance, *TPS* is involved in the synthesis of trehalose-6-phosphate. The interaction with *HXK* can promote the production of trehalose within cells, ensuring adequate energy supply and helping *B. dactyloides* cells maintain osmotic balance and reduce water loss and turgor pressure decline caused by salt stress [33]. *SPP* primarily converts sucrose-6-phosphate to sucrose, and its interaction with *HXK* can increase sucrose synthesis, thereby activating certain antioxidant enzymes and helping plants mitigate oxidative damage [34]. *AMY* degrades starch to produce glucose, and its interaction with *HXK* under salt stress can facilitate the rapid entry of glucose into glycolysis and other metabolic pathways, providing more energy to the plant [35]. SUS, which is involved in the synthesis and degradation of sucrose, can accelerate the breakdown of sucrose into glucose and fructose under salt stress [36]. After phosphorylation by *HXK*, these sugars release energy, affecting hormone levels such as auxin and cytokinin, and thus maintaining basic cellular physiological functions, enabling plants to better adapt to salt stress environments and sustain growth [37].

This study is the first to elucidate the number, distribution, and structural characteristics of the *HXK* gene family in the *B. dactyloides* genome under salt stress and to analyze their expression patterns over different time periods. These findings can provide important genetic information for future research on gene functions and molecular mechanisms and for the targeted improvement of salt tolerance in *B. dactyloides*.

## 4. Materials and Methods

### 4.1. Experimental Materials and Salt Stress Treatment

The experimental material was *Buchloe dactyloides* (Nutt.) Engelm., provided by the Chinese Academy of Forestry Sciences. Plump *B. dactyloides* seeds were surface-sterilized by soaking in 0.5% sodium hypochlorite for 5 min with occasional stirring to ensure thorough disinfection. After rinsing thoroughly, the seeds were soaked in 0.5% potassium nitrate for 24 h. Following another thorough rinse, the seeds were soaked in 2000 mg/L gibberellic acid for 24 h. After rinsing again and soaking in distilled water for 18 h, the seeds were washed and sown in seedling pots. The pots had an inner diameter of 12 cm and a depth of 11 cm, and the seedling substrate was nutrient soil. The plant growth conditions were maintained at a day/night temperature of 28 °C/24 °C. Twenty days after sowing, uniformly growing seedlings were selected and treated with 600 mM NaCl solution. Samples were collected at 0, 3, 6, 12, and 24 h after treatment, washed thoroughly, and divided into aboveground and underground parts. Each part was rapidly frozen in liquid nitrogen and stored at −80 °C for further analysis.

### 4.2. Identification and Physicochemical Property Analysis of B. dactyloides HXK Gene Family Members

The genome data of *B. dactyloides* was downloaded from the NCBI database (https://www.ncbi.nlm.nih.gov/). The protein sequences of the known hexokinase (*HXK*) gene family members from *Arabidopsis thaliana* (GenBank accession number: GCA_000001735.2), *Oryza sativa* (GenBank accession number: GCA_034140825.1), *Zea mays* (GenBank accession number: GCA_902167145.1), *Solanum lycopersicum* (GenBank accession number: GCA_036512215.2), and *Brachypodium distachyon* (GenBank accession number: GCA_000005505.4) were used as queries to perform Blastp comparisons against the full-length genome data of *B. dactyloides* to identify the *HXK* gene family members in *B. dactyloides*. All the websites were accessed on 2 November 2024. The physicochemical properties of the identified *B. dactyloides* HXK proteins, including the number of amino acids, relative molecular weight, isoelectric point (PI), and instability index, were predicted using the TBtools software. In order to ensure the accuracy and reliability of the results, we used the Plant—mPLoc subcellular localization prediction website (http://www.csbio.sjtu.edu.cn/bioinf/Cell-PLoc-2/, accessed on 11 November 2024) and the WoLF PSORT (https://wolfpsort.hgc.jp/, accessed on 13 November 2024) to predict *B. dactyloides* HXK proteins’ subcellular localization.

### 4.3. Phylogenetic Tree Construction and Salt Tolerance Expression Analysis of the B. dactyloides HXK Gene Family

To investigate the phylogenetic relationships within the *B. dactyloides HXK* gene family, multiple sequence alignments of the HXK protein sequences from *B. dactyloides*, *Arabidopsis thaliana*, *Oryza sativa*, *Zea mays*, *Solanum lycopersicum*, and *Brachypodium distachyon* were performed using MEGA X. The aligned sequences were used to construct a phylogenetic tree using the maximum likelihood (ML) method in TBtools software. The tree parameters were submitted to the ITOL website (https://itol.embl.de/, accessed on 14 November 2024) for beautification. The classification of the *B. dactyloides HXK* gene family was based on the classification method used for the rice *HXK* gene family [20]. This comparative analysis provides deeper insights into the evolutionary relationships and potential functional similarities of *HXK* genes across different species.

To understand the expression patterns of *BdHXK* genes in response to salt stress, the expression data of *BdHXK* genes under different salt stress treatments were extracted from the *B. dactyloides* transcriptome data. The TBtools software was used to generate a heatmap of the expression levels of key *BdHXK* genes in the roots at different time points of salt stress treatment.

### 4.4. Chromosomal Localization, Structure Analysis, Conserved Domain Analysis, Conserved Motif Analysis, and Cis-Acting Element Analysis of the B. dactyloides HXK Gene Family

The chromosomal positions of *HXK* genes in *B. dactyloides* were visualized using TBtools software. The coding sequence (CDS) of *B. dactyloides HXK* genes was used to obtain gene structure diagrams via TBtools software. The amino acid sequences of *B. dactyloides* HXK proteins were input into the NCBI conserved domain search program to identify the conserved domains they contain. The amino acid sequences were also uploaded to the MEME online tool (http://meme-suite.org/tools/meme accessed on 16 November 2024) to predict conserved motifs in the *BdHXK* gene family, with the number of motifs set to 10 [38]. The results were visualized using TBtools software.

The promoter sequences, 2000 bp upstream of the start codon of *B. dactyloides HXK* genes, were analyzed for cis-acting elements using the Plant CARE online tool (https://bioinformatics.psb.ugent.be/webtools/plantcare/, accessed on 17 November 2024) [39]. The analysis results were visualized using TBtools software.

### 4.5. Real-Time Quantitative PCR (RT-qPCR) Detection

The RNA was extracted using the TaKaRa RNA extraction kit, and cDNA was synthesized by reverse transcription(The kit was provided by Takara Bio Technology, Beijing Co., Ltd.). Primers were designed using Primer Premier 5.0 software (Premier Biosoft International is located in Palo Alto, CA, USA) and synthesized by Beijing RiboBio Tech Co., Ltd. (Beijing, China) (Supplementary Table S2). The PCR amplification was performed using 2× M5 HiPer SYBR Premix Es Taq (with Tli RNaseH) (Beijing PolyMedGene Biotech Co., Ltd., MF787). The reaction mixture was prepared as follows: 0.8 μL of cDNA template, 0.4 μL each of forward and reverse primers, 5 μL of 2× M5 HiPer SYBR Premix Es Taq, 0.2 μL of 50× ROX Reference Dye II, and 3.2 μL of ddH_2_O, bringing the total volume to 10 μL. The PCR reaction conditions were as follows: initial denaturation at 95 °C for 30 s; 40 cycles of 95 °C for 5 s and 60 °C for 30 s; followed by 95 °C for 5 s, 60 °C for 60 s, and 95 °C for 15 s; and a final extension at 8 °C for an indefinite period. Each gene was analyzed in triplicate. *DNAJ* was used as the reference gene [40]. The expression levels of the target genes were quantified using the 2^−ΔΔCt^ method [41], with the expression level of *DNAJ* at 0 h set to 1. The expression levels of other genes and at different time points were compared to this reference for quantification.

### 4.6. WGCNA Analysis of the B. dactyloides HXK Gene Family

To determine the functions and evolutionary relationships of the BdHXKs genes, we analyzed the transcriptome data of the key genes. Firstly, the WGCNA network was constructed using the one-step method [42]. The FPKN values were input, and the genes with similar expression patterns in the output were grouped into one module. The key *BdHXK* genes were selected as the hub genes to construct a plant co-expression network. The network was visualized using Cytoscape v3.10.0 [43].

### 4.7. Data Analysis

Data analysis was performed using Excel 2010. One-way ANOVA was conducted using IBM SPSS Statistics 27 to assess the significance of differences (*p* < 0.05). The Duncan test was used to evaluate the significance of the differences. Bar charts were created using Origin 2021 for visual representation of the data.

## 5. Conclusions

In this study, 25 hexokinase (HXK) genes, designated *BdHXK1* to *BdHXK25*, were identified from the *Buchloe dactyloides* genome. The 25 *BdHXK* genes are unevenly distributed across 8 chromosomes.

Phylogenetic analysis divided the *BdHXK* family into three subfamilies, with the closest evolutionary relationships to rice *HXK*s. Promoter sequence analysis revealed the presence of stress-responsive elements, indicating their involvement in responses to abiotic stresses and hormone signaling. Relative expression analysis showed significant upregulation of *BdHXK5*, *BdHXK6*, *BdHXK7*, and *BdHXK23* under salt stress, suggesting their potential as key regulators of salt tolerance in *B. dactyloides*. This provides a potential gene target for the screening of salt-tolerant crops, and the subsequent focus on crop varieties with these key regulatory factors or their related expression characteristics can help the screening of salt-tolerant crops.

These results enhance our understanding of the *BdHXK* gene family’s role in salt stress response and provide a foundation for further research on sugar metabolism, energy supply, and signal transduction. Future studies can focus on elucidating the specific functions and interactions of individual *BdHXK* members to gain more comprehensive insight into the growth and development of *B. dactyloides* and its resistance to abiotic stresses. It is expected to promote the cultivation of new strains of ecological restoration grass with more salt resistance and better growth and development and help the improvement and ecological restoration of saline–alkali land.

## Data Availability

The datasets presented in this study can be found in online repositories. The names of the repository/repositories and accession number(s) can be found at: https://www.ncbi.nlm.nih.gov/, 2 November 2024, GCA_000001735.2, GCA_034140825.1, GCA_902167145.1, GCA_036512215.2, GCA_000005505.4.

## Supplementary Materials

The following supporting information can be downloaded at: https://www.mdpi.com/article/10.3390/ijms26020838/s1.
