# Supplementary material for: Identification of HXK Gene Family and Expression Analysis of Salt Tolerance in Buchloe dactyloides
Source: Int J Mol Sci. 2025 Jan 20;26(2):838. doi: 10.3390/ijms26020838 (PMC11765778; doi:10.3390/ijms26020838)
Supplement: Supplementary file 1 [file ijms-26-00838-s001.zip › Appendix/Supplementary Table S1.pdf]

Gene name	Sequence ID	Number of Amino Acid	Molecular Weight	Theoretical pI	Instability Index	Aliphatic Index	Grand Average of Hydropathicity	subcellular localization
BdHXK1	Bd30g0540.1	441	47372.95	5.03	36.92	97.80	-0.044	Chloroplast. Mitochondrion.
BdHXK2	Bd32g0501.1	475	50810.94	5.14	36.76	98.63	0.003	Mitochondrion.
BdHXK3	Bd29g0518.1	475	50754.83	5.18	34.24	97.01	0.004	Mitochondrion.
BdHXK4	Bd32g0500.1	466	49956.88	5.31	36.56	94.87	-0.044	Chloroplast. Mitochondrion.
BdHXK5	Bd31g0536.1	466	49923.81	5.30	36.43	94.66	-0.040	Chloroplast. Mitochondrion.
BdHXK6	Bd29g0517.1	466	50019.01	5.45	37.38	94.66	-0.034	Chloroplast.
BdHXK7	Bd30g0539.1	465	49927.77	5.30	36.94	93.61	-0.058	Chloroplast.
BdHXK8	Bd11g0146.1	498	53332.78	5.76	37.83	99.32	0.043	Mitochondrion.
BdHXK9	Bd10g0158.1	498	53267.66	5.65	37.68	98.53	0.049	Mitochondrion.
BdHXK10	Bd12g0161.1	498	53394.81	5.76	38.39	98.53	0.040	Mitochondrion.
BdHXK11	Bd31g1617.1	509	55070.20	5.82	33.60	97.94	0.006	Chloroplast. Mitochondrion.
BdHXK12	Bd29g1610.1	509	55055.16	5.78	33.41	96.97	0.003	Chloroplast. Mitochondrion.
BdHXK13	Bd32g1598.1	509	55044.18	5.73	34.25	97.94	0.015	Chloroplast. Mitochondrion.
BdHXK14	Bd12g1184.1	508	55472.49	5.96	34.07	94.27	-0.113	Chloroplast. Mitochondrion.
BdHXK15	Bd09g1226.1	508	55406.34	5.96	34.29	93.35	-0.121	Chloroplast. Mitochondrion.
BdHXK16	Bd11g1170.1	513	56093.17	5.96	34.31	92.63	-0.110	Chloroplast. Mitochondrion.
BdHXK17	Bd10g1221.1	523	57141.43	5.96	36.83	95.14	-0.081	Chloroplast. Mitochondrion.
BdHXK18	Bd11g2346.1	480	52089.32	5.44	34.50	90.46	-0.152	Chloroplast.
BdHXK19	Bd12g2313.1	483	52373.66	5.44	36.51	91.33	-0.133	Mitochondrion.
BdHXK20	Bd09g2417.1	483	52329.69	5.59	35.68	91.74	-0.140	Mitochondrion.
BdHXK21	Bd10g2452.1	479	52006.19	5.44	34.95	89.65	-0.172	Chloroplast.
BdHXK22	Bd30g0957.1	526	57442.40	5.81	46.38	103.84	0.167	Mitochondrion.
BdHXK23	Bd32g0902.1	531	58056.96	6.02	44.89	101.21	0.122	Mitochondrion.
BdHXK24	Bd31g0911.1	500	54091.13	5.57	45.83	100.28	0.088	Mitochondrion.
BdHXK25	Bd29g0916.1	500	54062.22	5.79	45.39	101.06	0.109	Mitochondrion.
