# Supplementary material for: Identification of HXK Gene Family and Expression Analysis of Salt Tolerance in Buchloe dactyloides
Source: Int J Mol Sci. 2025 Jan 20;26(2):838. doi: 10.3390/ijms26020838 (PMC11765778; doi:10.3390/ijms26020838)
Supplement: Supplementary file 1 [file ijms-26-00838-s001.zip › Appendix/Supplementary Table S2.pdf]

Gene name	Forward primer（5' to 3'）	Reverse primer（5' to 3'）
BdHXK1	GATTCGTTCTGCGGATTG	CCGTCTCGTAATGATGTCG
BdHXK2	CGTCAGTGCTGGCAGAGTT	TGAGCGTGCCCGAGG
BdHXK3	GCAACAAGCGGAGGGAACT	CTGGGCGACGACGTTCTC
BdHXK4	GGACGCGATTCGTCCTG	CCTCGTGACGATATCGCAG
BdHXK5	GGACGTCTCCGCGATGC	CTACCATCTTCCTCGTTTCAAGG
BdHXK6	GGCGAGGACGTTGTCGG	GGCGACGACGCAGAGGT
BdHXK7	CCGGGAGGTACAACGACG	GGCAGGTGGTCCAGCTTG
BdHXK8	CGGAAATGAAGAAGGTGTAT	GTTCCTCAGGGATGGGT
BdHXK9	CAGGGATTTACCTTGGG	GTCCGGTATCCTCAAATG
BdHXK10	GCCTGCGCCAGGTCGT	TACGCCTTCTTCATTTCCGC
BdHXK11	TCCGCGGCGACATCC	TTTGGTGCACTGGGAATGAG
BdHXK12	GACGATTTCCATCTCCCTG	ACTTTCATATCAAGCCCCTG
BdHXK13	TTTACGCACTGGATCTTGGA	CTTGACAACACGTTTCTCCC
BdHXK14	AAGTGACAGTTTCCAGACGC	TGATGACAACCGACGAGG
BdHXK15	GTGACACTTTCCAGACGC	CTGGGAATGTGAGGCC
BdHXK16	TTCCATGGAACTGTTCGATT	AGCCCTTGGTCCACTTAATT
BdHXK17	ACCTATGGTGTCTGAATTTATCC	AGCGCCTAGGTGTTTGAG
BdHXK18	CTGTCGATGGAGGACTGTT	ATAGATACTGACTCTGCCGC
BdHXK19	GCAATCTTCAGTTTTCGGC	TAGCCGAGCCGCCC
BdHXK20	GGCAGCAGAGTCAGTAT	CTCTTGCCCACTCTTAG
BdHXK21	TCAGTTACTGCTATCGATGG	CCATCTTCTGCATGCTTC
BdHXK22	GCACACGAGATTGCTATGAC	AATCCGTAGCAGAACCGAAT
BdHXK23	CGATCGAAGGCGGTCTGTAC	GTTGTCGACGAGTATGACGCTG
BdHXK24	GGAAAGATGTTGCTCAGTGC	ATCATAATAGTGACCCAAAGCC
BdHXK25	AAAGCTCCATATCCTCAGGGT	ATAATAGTGCCCCAAAGCCA
DNAJ	ACACGGAACAATGGTACACT	TCCCGATCCTTCCTATGCTC
