# Supplementary material for: Identification of HXK Gene Family and Expression Analysis of Salt Tolerance in Buchloe dactyloides
Source: Int J Mol Sci. 2025 Jan 20;26(2):838. doi: 10.3390/ijms26020838 (PMC11765778; doi:10.3390/ijms26020838)
Supplement: Supplementary file 1 [file ijms-26-00838-s001.zip › Appendix/Supplementary Table S3.pdf]

Interaction Node	Interacting Gene ID	Gene Length	KO ID	KO Description	Swissprot ID	Swissprot Description
TPS10-2	Cluster-36265.82292	5642	K16055	trehalose 6-phosphate synthase	O80738	Probable alpha,alpha-trehalose-phosphate synthase 10
SPP3	Cluster-36265.84157	4062	K07024	sucrose-6-phosphatase	A3AZW5	Probable sucrose-phosphatase 3
HXK7-2	Cluster-36265.73530	1551	K00844	hexokinase	Q1WM16	Hexokinase-7
TPS10-1	Cluster-36265.85673	5060	K16055	trehalose 6-phosphate synthase	O80738	Probable alpha,alpha-trehalose-phosphate synthase 10
TPS6-1	Cluster-36265.85411	2312	K16055	trehalose 6-phosphate synthase	Q94AH8	Alpha,alpha-trehalose-phosphate synthase 6
TPS11-1	Cluster-36265.85680	2807	K16055	trehalose 6-phosphate synthase	Q9ZV48	Probable alpha,alpha-trehalose-phosphate synthase 11
HXK7-1	Cluster-36265.83862	1573	K00844	hexokinase	Q1WM16	Hexokinase-7
AMY3E	Cluster-36265.74242	1304	K01176	alpha-amylase	P27934	Alpha-amylase isozyme 3E
SUS4	Cluster-36265.94048	585	K00695	sucrose synthase	Q10LP5	Sucrose synthase 4
FRK1	Cluster-36265.79551	3382	K00847	fructokinase	Q6XZ79	Fructokinase-1
TPS11-2	Cluster-36265.82402	1203	K16055	trehalose 6-phosphate synthase	Q9ZV48	Probable alpha,alpha-trehalose-phosphate synthase 11
TPS6-2	Cluster-36265.90047	1345	K16055	trehalose 6-phosphate synthase	Q94AH8	Alpha,alpha-trehalose-phosphate synthase 6
bglX	Cluster-36265.64808	1554	K05349	beta-glucosidase	Q23892	Lysosomal beta glucosidase
GH3B	Cluster-36265.64809	1087	K05349	beta-glucosidase	A7LXU3	Beta-glucosidase BoGH3B
