# Supplementary figures and images for: Identification of HXK Gene Family and Expression Analysis of Salt Tolerance in Buchloe dactyloides
Source: Int J Mol Sci. 2025 Jan 20;26(2):838. doi: 10.3390/ijms26020838 (PMC11765778; doi:10.3390/ijms26020838)

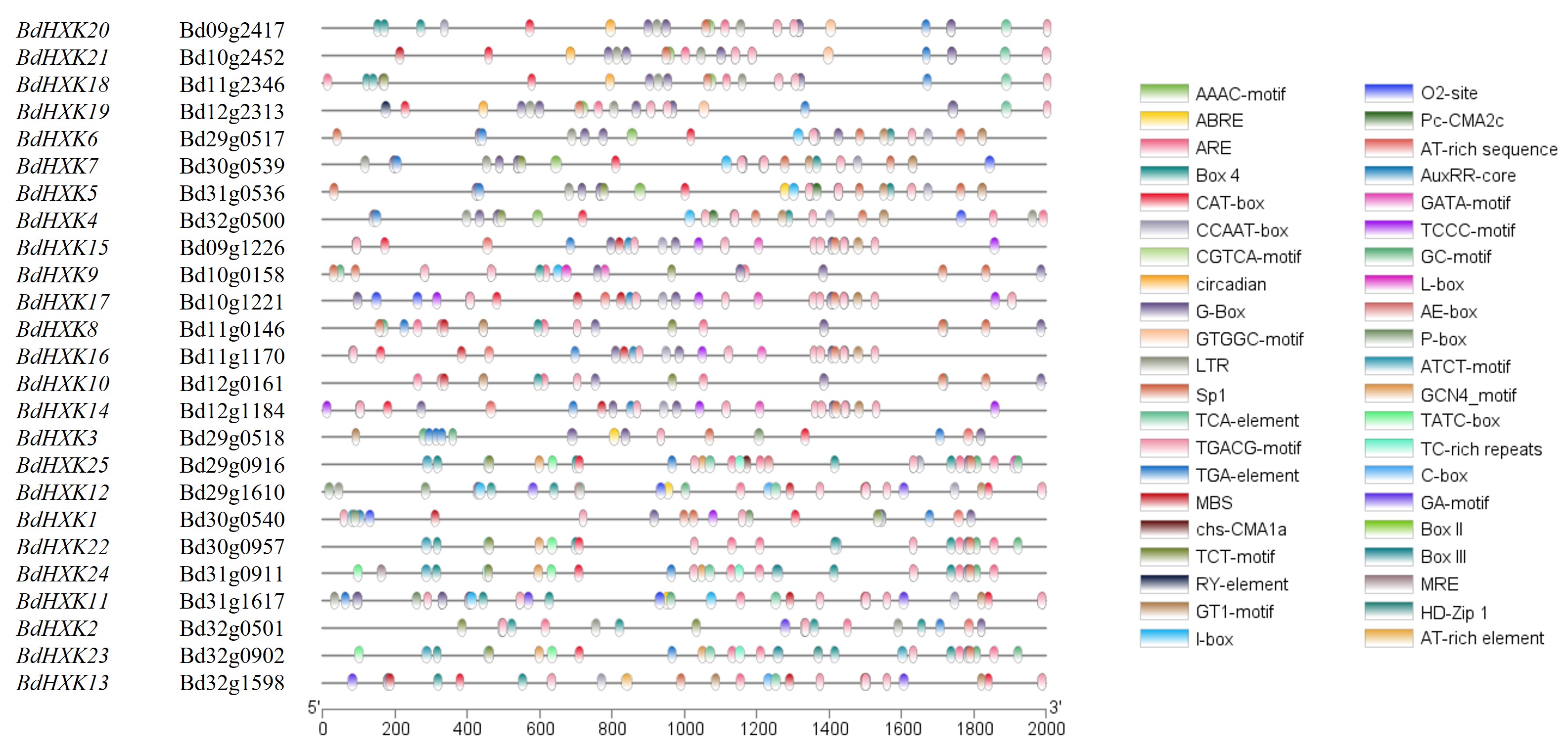

Supplement: Supplementary file 1 [file ijms-26-00838-s001.zip › Appendix/Supplementary Figure 1.jpg]
